# Corrosion Behavior of Ni-P/Cu Catalyst in Optimization of Electroplating Process Inside the NaBH_4_ Seawater Fuel Cell

**DOI:** 10.3390/ma19112178

**Published:** 2026-05-22

**Authors:** Li Sun, Ruihan Shen, Fenglin Han, Shuchang Zhang, Hongzhou Zhang, Yongsheng Wei

**Affiliations:** 1 School of Marine Science and Technology, Northwestern Polytechnical University, Xi’an 710072, China; sunli25@nwpu.edu.cn (L.S.); shenrh@mail.nwpu.edu.cn (R.S.); 1261031106@mail.nwpu.edu.cn (F.H.); 2 Ocean Institute, Northwestern Polytechnical University, Xi’an 710072, China; 13868863055@163.com (S.Z.); 15327104349@163.com (H.Z.); 3 Shenzhen Research Institute, Northwestern Polytechnical University, Shenzhen 518057, China; 4 Yangtze River Delta Research Institute, Northwestern Polytechnical University, Taicang 215400, China

**Keywords:** corrosion mechanism, hydrogen energy, Ni-P/Cu catalyst, fuel cell

## Abstract

Lamellar structure Ni-P catalysts were prepared on copper by the electrochemical deposition method for the hydrolysis of NaBH_4_ solution. Voltage, time and temperature are key variables in the electroplating process, affecting the corrosion performance of the catalyst. The results show that as the deposition voltage (4–7 V) increases, the corrosion resistance of Ni-P at first is enhanced and then decreases, peaking at 5 V due to a more complete structure. Electroplating time and temperature affect the deposition of the nickel-phosphorus catalyst and then the corrosion resistance of the catalyst. Prolonged time and elevated temperature cause holes and cracks, degrading corrosion resistance. Therefore, a mild electroplating environment is preferred. The optimal electroplating temperature and time are 30 °C and 3 min, respectively. The polarization curve test shows that the Ni-P catalyst is greatly influenced by seawater temperature and chloride ion concentration in the actual service process, that the chloride ion is the dominant factor, and that the corrosion rate increases exponentially. Moreover, Ni-P/Cu catalysts mainly undergo localized corrosion and dissolution. Combined with Scanning Electron Microscope (SEM) and Energy Dispersive Spectrometer (EDS) analyses, the corrosion mechanism in seawater was systematically discussed.

## 1. Introduction

Hydrogen energy is attracting increasing attention from researchers all around the world because of its high calorific value and environmental benignity [1]. However, due to the lack of efficient and stable hydrogen production methods [2], the large-scale application of hydrogen is seriously limited. In addition, the current ways to store hydrogen are gas hydrogen storage and hydraulic hydrogen storage by tank [3], which invariably require a high-pressure, heat-insulation and low-temperature environment [4]. In this case, the hydrogen tank has a high maintenance cost and is always on the verge of leakage and explosion [5]. This is one of the important reasons why hydrogen energy is difficult to popularize, although the hydrogen energy density is higher than that of lithium batteries [6], and it is easier to obtain than petrochemical resources [7]. In order to solve the current dilemma, various solid hydrogen storage materials, such as NaBH_4_ [8,9], KBH_4_ [10], LiBH_4_ [11], NaAlH_4_ and MgH_2_, came into being [12,13].

Sodium borohydride (NaBH_4_) is regarded as one of the important hydrogen storage materials because of its high hydrogen storage density, nonpoisonous nature and low price. It can be seen from the equation of the hydrolysis of NaBH_4_ that 4 mol of hydrogen can be obtained from 1 mol of NaBH_4_, including 2 mol of hydrogen in water [14,15,16]. In view of this, the actual hydrogen storage density is as high as 21.6 wt.%, and no volatile outgrowth is produced [17]. The high-purity humidified hydrogen can be used as a raw source for NaBH_4_ fuel cells. Hydrogen production via the hydrolysis of NaBH_4_ at room temperature is a potential method. However, the overpotential induced by the hydrogen evolution reaction (HER) needs precious metal catalysts or their compounds (Ru, Pt, Au, Rd and Rh) [18] due to the natural catalytic activity, which hinders the development of hydrogen production technology by water electrolysis because of high cost and the lack of resources [19,20,21]. Therefore, it is urgent to develop an efficient, cheap and durable catalyst material.

In recent exploration, Co and Ni non-noble [22] transition metal-based catalysts have received attention from researchers. Although these catalysts exhibit excellent performance in catalyzing NaBH_4_ hydrolysis, their stability is far less satisfactory than that of noble metal catalysts. Yu et al. prepared a PtNi alloy catalyst, and its HGR loading was as high as 10,164.3 mL min^−1^·g^−1^. After eight cycles, the catalyst still retained 87.8% activity of the initial state [23]. In contrast, Luo et al. prepared a CoNi layered hydroxide nanosheet composite linked by multi-walled carbon nanotubes, and the optimum HGR reached 5167.72 mL min^−1^·g^−1^, but after 10 cycles, the HGR decreased to 75.4% of the initial value [24]. Wang et al. employed chemical vapor deposition to fabricate Co-P catalyst supported on CNT-Ni foam surface, and the detected HGR reached up to 2430 mL min^−1^·g^−1^ but decreased to 74% after eight cycles compared with the initial level [25]. Overall, researchers are exploring higher HGR, and the experimental parameters meet the needs of most equipment, but the stability or durability of catalysts is rarely considered [26,27,28]. HGR is only an index of fuel cells, and the durability related to service life is also worthy of attention. In the marine environment, NaBH_4_ is directly injected into seawater for hydrolysis [29]. In this process, seawater, regarded as a unique solvent, will cause severe corrosion to the metal-based catalyst [30]. Thus, the development of an anticorrosive and non-shedding catalyst for the NaBH_4_ hydrolysis is key for the hydrogen generation of fuel cells [31,32].

In this paper, Cu sheet-supported Ni-P catalysts were prepared by electroless plating in a NaBH_4_ fuel cell. The corrosion resistance of Ni-P/Cu catalysts with different deposited voltages (V), times (t) and temperatures (T) was adequately studied. In addition, experiments on seawater temperature, chloride ion accelerated corrosion and types of chloride ion corrosion were also carried out to illustrate the accelerated corrosion mechanism of the catalyst in the NaBH_4_ fuel cell. For this purpose, electrochemical technologies, i.e., potentiodynamic polarization, electrochemical impedance spectroscopy, and SEM analysis, were employed to study the corrosion behavior of the Ni-P/Cu catalysts in the NaBH_4_ seawater fuel cell.

## 2. Experimental

### 2.1. Catalyst Preparation and Characterization

It is well established that the preparation process of a catalyst significantly impacts its performance. In our preliminary work, we explored the optimization of parameters such as pH [33] and ion concentration [8]—factors closely tied to the solution system. Electroplating process parameters constitute another critical set of influencing factors—specifically voltage, duration, and temperature—and these form the core subject of this study. The chemical reagents included nickel sulfate dihydrate (NiSO_4_·2H_2_O, AR), sodium hypophosphite monohydrate (NaH_2_PO_2_·H_2_O, AR), sodium citrate dihydrate (C_6_H_5_Na_3_O_7_·2H_2_O, AR), boric acid (H_3_BO_3_, AR), sodium hydroxide (NaOH, AR), and sodium borohydride (NaBH_4_, AR), all of which were purchased from Sinopharm Chemical Reagent Co., Ltd., (Shanghai, China). and used without further purification. To investigate the effects of different catalyst preparation conditions on the microstructure and properties of the catalysts, the reagent dosage, the deposition voltages, times, temperatures and pH parameters were listed in Table 1. The Ni-P/Cu ternary catalyst was prepared on a copper substrate by electrodeposition. The substrate used in the experiment was a copper sheet with dimensions of 5 × 5 × 2 mm. The counter electrode in the electrodeposition process was a graphite plate. During electrodeposition, the voltages applied between the copper and graphite plates were 4 V, 5 V, 6 V, and 7 V, respectively.

Prior to electrodeposition, the copper sheet was ultrasonically cleaned in acetone, ethanol, and deionized water for 10 min each to remove surface oils and impurities. The Ni-P/Cu catalyst was then prepared by electrodeposition. First, 0.79 g of NaH_2_PO_2_·H_2_O was dissolved in 100 mL of deionized water and stirred at 600 rpm for 30 min using a magnetic stirrer from Changzhou Surui Instrument Co., Ltd., (Changzhou, China). Next, 1.03 g of C_6_H_5_Na_3_O_7_·2H_2_O (serving as a complexing agent to enhance the conductivity and deposition efficiency of the plating solution) and 2.48 g of H_3_BO_3_ (used to adjust the solution pH to acidic conditions and stabilize the electrodeposition system) were added. Finally, 0.95 g of NiSO_4_·2H_2_O was added, and the mixture was stirred continuously for 1 h. The solution was then allowed to stand for 3 h to confirm that no precipitate was formed. Detailed reagents and experimental conditions are listed in Table 1.

### 2.2. Corrosion Resistance Test

Electrochemical tests were conducted using a CHI660E electrochemical workstation from Shanghai Chenhua Instrument Co., Ltd., (Shanghai, China). with a three-electrode system. The working electrode was a copper sheet coated with a Ni-P/Cu catalyst (exposed area of 1 cm^2^), the counter electrode was a graphite rod, and the reference electrode was a saturated calomel electrode (SCE). A mixed solution of 2 mol/L NaOH and 2 mol/L NaBH_4_ was used to simulate the borohydride fuel cell environment. The electrolyte consisted of simulated seawater. Prior to testing, the working electrode (Ni-P/Cu catalyst) was immersed in the electrolyte for 30 min, and the open circuit potential (OCP) variation was recorded for 1200 s to ensure the electrode/electrolyte interface reached a quasi-steady state. Potentiodynamic polarization curves were initiated at −0.2 V (vs. Ag/AgCl) below the OCP, with a scan rate of 0.1667 mV/s. The corrosion potential (E_corr_) and corrosion current density (I_corr_) were determined by fitting the Tafel curves to quantitatively evaluate the catalyst’s corrosion rate. Electrochemical impedance spectroscopy (EIS) was performed under stable OCP conditions, with a frequency range of 0.01 Hz–100 kHz and an AC signal amplitude of 5 mV. Corrosion kinetics and interfacial impedance characteristics of the electrode surface were analyzed by fitting Nyquist and Bode plots. The EIS data were modeled using an equivalent circuit. In linear sweep voltammetry (LSV) testing, the scan range was −0.5 V to 0.1 V, and the scan rate was 2 mV·s^−1^. After the electrochemical tests, SEM was employed to observe the morphology and structure of the Ni-P/Cu catalyst, and EDS was used to determine the optimal electroplating parameters for the catalyst. The instruments used for SEM and EDS testing were the Zeiss Gemini SEM 460, from Carl Zeiss AG (Oberkochen, Germany).

## 3. Results and Discussion

### 3.1. Composition and Structure of Catalyst

**Figure 1:**
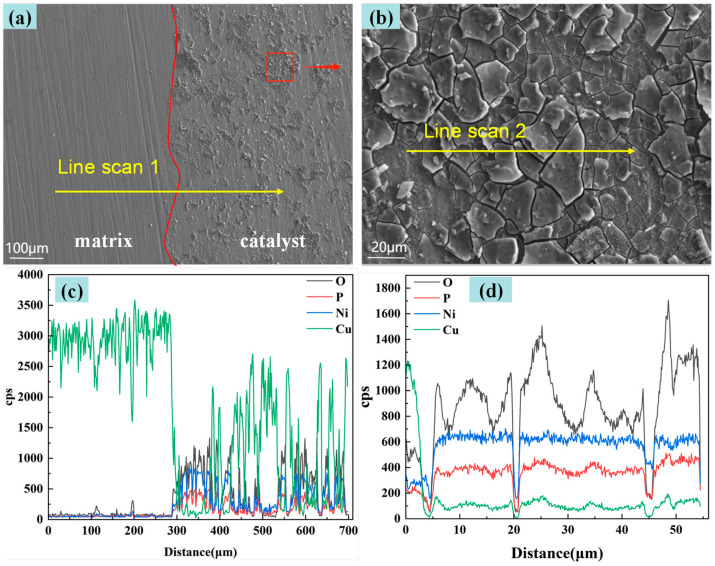
(**a**) The junction between the copper and Ni-P/Cu, (**b**) enlarged morphology of the Ni-P/Cu catalyst, (**c**) line scan 1 of the surface elements, and (**d**) line scan 2 of the surface elements. The preparation conditions of the Ni-P/Cu catalyst are 20 °C, 1 min, 5 V.

Figure 1 illustrates the typical morphology and elemental distribution of the catalyst coating prepared under conditions of 20 °C, 1 min, and 5 V. Figure 1a shows the morphology of the copper sheet before and after electrodeposition. As clearly observed, the copper substrate surface on the left side (without catalyst plating) is relatively smooth and flat with a distinct texture. This indicates that no other metal particles were deposited on the copper sheet in the absence of Ni and P elements. It can be inferred that the electrooxidation efficiency of sodium borohydride fuel cells is relatively low due to the lack of catalytic activity [19,31]. In contrast to the right side of Figure 1a, it is clearly observed that the catalyst attached to the copper surface significantly altered its morphology, demonstrating that the electrodeposition method effectively enables catalyst deposition on copper. The electrodeposition process of Ni and P elements proceeded relatively completely, confirming the successful deposition of the prepared Ni-P/Cu catalyst on the copper sheet. The catalyst region in Figure 1a was further magnified, as shown in Figure 1b. The catalyst exhibited a scale-like aggregated morphology attached to the copper surface, with an uneven layer thickness and large cracks between the catalyst flakes. These cracks are attributed to the uneven thickness of the catalyst layer, where internal stresses generated within the coating caused significant gaps. It is likely that these cracks would accelerate catalyst detachment. Therefore, optimizing deposition voltage, time, and temperature is crucial for catalyst preparation. To more clearly illustrate the variation in catalyst composition, Figure 1c,d present the compositional analysis of the Ni-P/Cu catalyst. It is distinctly observed that, using the red line in Figure 1a as the dividing line, the curve corresponding to the left side in Figure 1c indicates an extremely high Cu content, with other elements nearly absent from the copper surface; the curve corresponding to the right side shows significant increases in Ni and P contents. This is because, under the effect of electroless plating, the Ni-P catalyst covered the copper substrate surface. Figure 1d shows the line scan diagram of the elemental composition of the Ni-P/Cu catalyst. As can be seen from the figure, copper content is the lowest, which is because the coating covers the substrate surface. The increased Ni and P content indicates successful coating preparation. The increased oxygen content indicates that metallic copper and nickel underwent natural oxidation in air.

**Figure 2:**
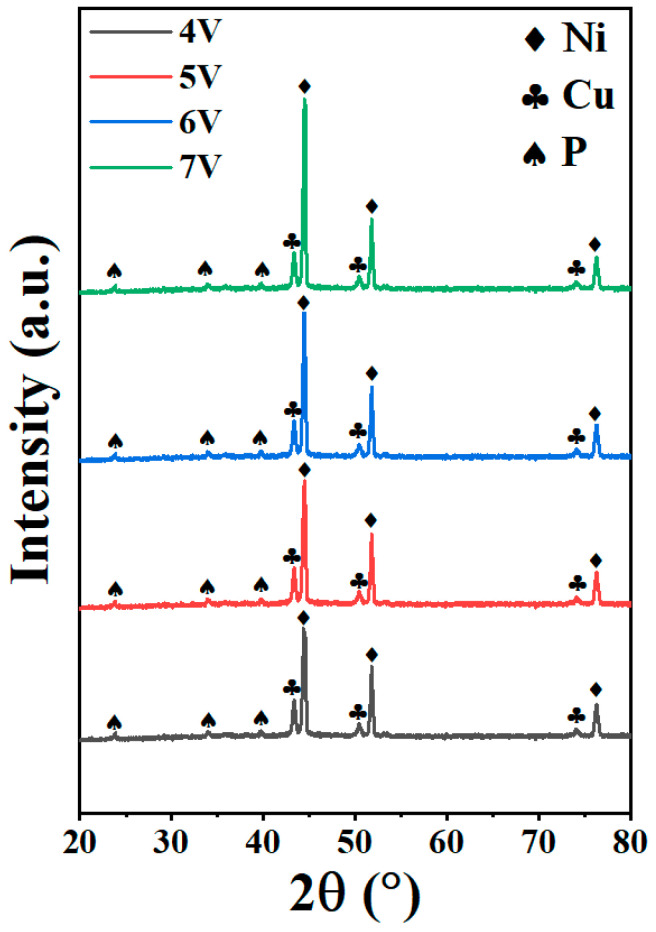
The XRD patterns of Ni-P/Cu coating at different voltages.

Figure 2 shows the XRD patterns of Ni-P coatings on the copper surface at different potentials. It is observed that the spectrum shows the peaks of Ni, Cu and P, which indicates that the Ni-P catalyst has been successfully prepared. The peak value of the Ni element is the highest, indicating that the catalyst is mainly composed of Ni. With the increase in potential, the Ni content gradually increases and the coating thickens, which is consistent with our previous research [8]. Combined with the analysis in Figure 1, Ni-P coating is successfully prepared on a copper sheet by electrochemical deposition.

**Figure 3:**
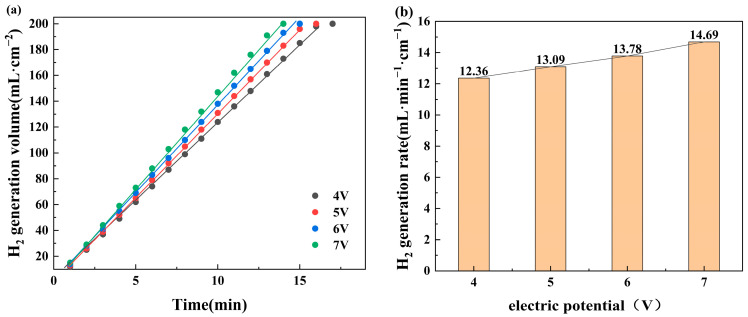
(**a**) Hydrogen generation kinetics from the hydrolysis of NaBH_4_ solution catalyzed by Ni-P/Cu catalysts at different voltages, (**b**) hydrogen generation rate. The electroplating temperature and time are 30 °C and 3 min.

Figure 3 exhibits the kinetic characteristics of hydrogen evolution from NaBH4 solution catalyzed by Ni-P/Cu catalysts prepared at different voltages. It is seen that with increasing deposition voltage, the rate of hydrogen evolution increases gradually (Figure 3a). According to the curve slope of hydrogen production and time, the hydrogen generation rates (i.e., the catalytic activity) at 4 V, 5 V, 6 V and 7 V are 12.36 mL·min^−1^·cm^−1^, 13.09 mL·min^−1^·cm^−1^, 13.78 mL·min^−1^·cm^−1^ and 14.69 mL·min^−1^·cm^−1,^ respectively, as shown in Figure 3b. However, the higher the activity of the catalyst, the worse the corrosion resistance. The corrosion resistance test of the Ni-P/Cu catalyst will be discussed below.

**Figure 4:**
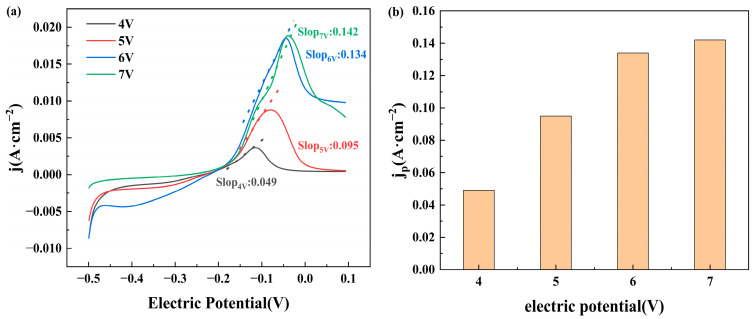
(**a**) LSV tests corresponding to Ni-P/Cu catalysts at different voltages. The dashed lines represent linear fitting lines used to determine the slopes of the anodic polarization peaks in the LSV curves, (**b**) peak current density of LSV curve. The electroplating temperature and time are 30 °C and 3 min.

Figure 4 shows the LSV tests corresponding to Ni-P/Cu catalysts at different voltages. With the increase in deposition voltage, the positive slope of the oxidation peak gradually increases (Figure 4a), and the peak value of the maximum current density also gradually increases (Figure 4b), indicating that the Ni-P/Cu catalyst’s reactivity is enhanced. This trend is consistent with Figure 3, further illustrating that as the deposition voltage increases, the amount of Ni-P/Cu catalyst deposited on the copper surface increases, and the catalytic activity is enhanced. However, this will lead to an increased corrosion rate and a shortened lifespan for Ni-P catalysts.

### 3.2. Electrochemical Corrosion Research

It is well known that the corrosion resistance of catalysts in hydrogen fuel cells is critically important, as it directly affects the service life of the cells [8,32]. In practical applications, the catalyst is directly exposed to a mixed solution of seawater and sodium borohydride. Seawater serves as the primary electrolyte for hydrogen fuel cells and is widely used in deep-sea equipment. Figure 2 illustrates the evolution of the open circuit potential (OCP) of Ni-P/Cu catalysts during operation in hydrogen fuel cells under different preparation parameters, including deposition voltage, time, and temperature.

**Figure 5:**
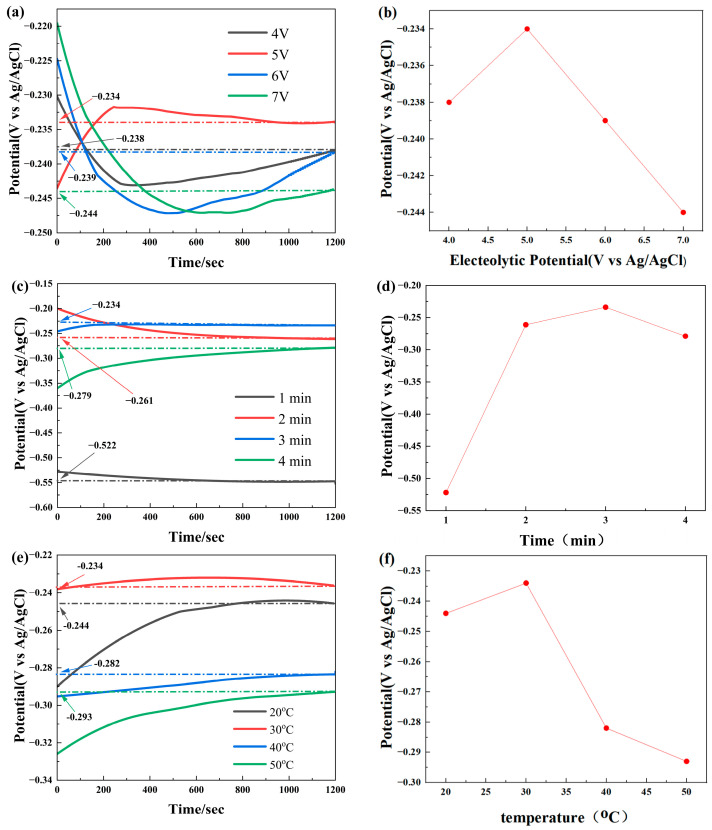
The dependences of OCP of Ni-P/Cu catalysts on different preparation conditions. (**a**,**b**) Voltage, when electroplating temperature and time are 30 °C and 3 min; (**c**,**d**) time, when electroplating temperature and voltage are 30 °C and 5 V; (**e**,**f**) temperature, when electroplating voltage and time are 5 V and 3 min.

As shown in Figure 5a,b, when the electrodeposition voltages were 4 V, 6 V, and 7 V, the open circuit potential (OCP) initially shifted negatively and then positively. This is the opposite of the 5 V state, where the OCP initially increased and then stabilized. This change is due to the fact that the Ni-P coating prepared at 5 V has the highest open circuit potential (OCP), thus exhibiting thermodynamic stability during corrosion and optimal corrosion resistance. It is generally accepted that higher OCP values indicate lower electrochemical activity and a reduced tendency for activation corrosion of the metal [34,35,36]. Therefore, as the deposition voltage increased (4–5 V), the Ni-P catalyst gradually covered the copper surface, forming a dense and smooth “coating” that caused the OCP to shift positively. However, when the deposition voltage further increased (5–7 V), the “coating” became uneven in thickness, exhibiting cracks and brittleness due to internal stress (Figure 1b), leading to corrosion of the Ni-P catalyst by seawater and accelerated corrosion damage, resulting in a rapid decrease in OCP. It should be noted that all OCP values were lower than the electrode voltage of copper (+0.34 V), which further demonstrates that the copper substrate itself possesses higher corrosion resistance than the Ni-P catalyst.

Figure 5c,d illustrate the variation in OCP for Ni-P catalysts with deposition time. It can be observed that the OCP initially evolves slowly and then stabilizes, indicating that the Ni-P/Cu catalyst reaches dissolution equilibrium within the cell. When the electrodeposition time is 3 min, the OCP stabilizes at approximately −0.25 V. This demonstrates that electrodeposition time significantly influences catalyst performance, and the observed trend suggests the existence of an optimal electrodeposition time. The OCP of Ni-P catalysts also changes markedly with increasing deposition temperature (Figure 5e,f), showing a decreasing trend as temperature rises. Therefore, selecting an appropriate electrodeposition temperature is crucial for catalyst preparation and activity. Consequently, we conclude that excessively long electrodeposition times and excessively high temperatures promote electrochemical corrosion of the Ni-P catalyst, which substantially reduces the service life of Ni-P catalysts. It should be noted that OCP variation reflects a thermodynamic evolution process, whereas the actual corrosion pathways and mechanisms require investigation through kinetic experiments.

**Figure 6:**
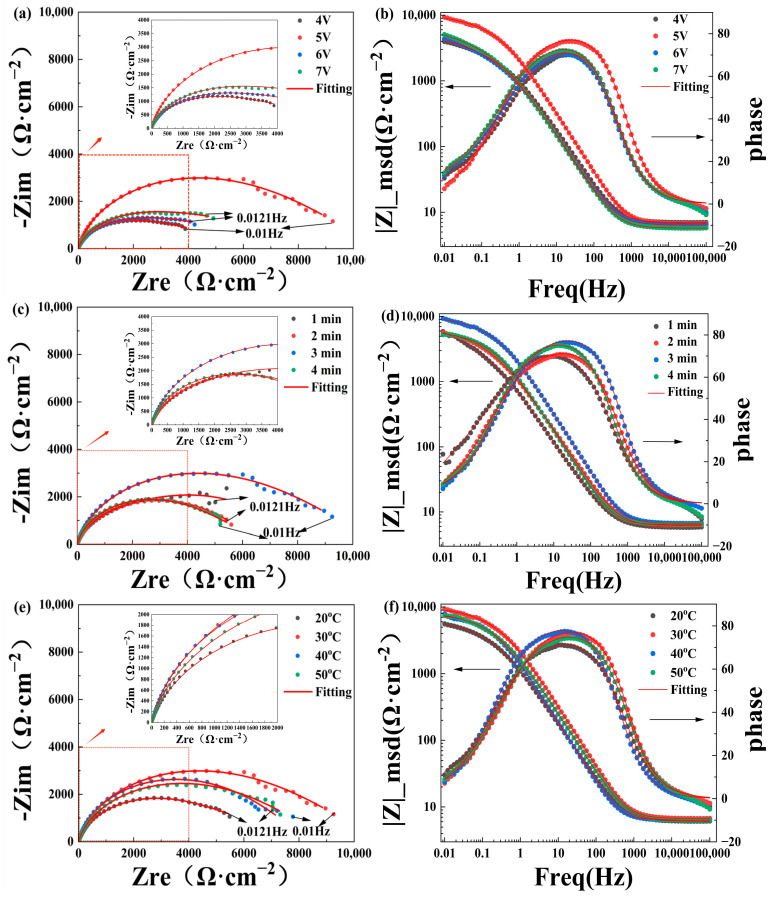
The Nyquist plots and Bode plots of Ni-P/Cu catalysts prepared under different conditions: (**a**,**b**) voltage, when electroplating temperature and time are 30 °C and 3 min; (**c**,**d**) time, when electroplating temperature and voltage are 30 °C and 5 V; (**e**,**f**) temperature, when electroplating voltage and time are 5 V and 3 min.

Figure 6 presents the electrochemical impedance spectroscopy (EIS) of Ni-P catalysts under various electrodeposition conditions. The impedance modulus plots typically provide valuable information regarding corrosion progression. The similar shapes of all curves indicate consistent corrosion processes and mechanisms. The Nyquist plot (Figure 6a) shows that as the deposition voltage increases, the impedance arc initially grows larger, reaching a maximum at 5 V. Furthermore, the corresponding phase angle plot (Figure 6b) reveals that when the voltage increases to 5 V, the maximum phase angle shifts significantly toward the low-frequency region; however, with further increases in voltage, the phase angle gradually decreases. This suggests that as the electrodeposition voltage rises, the Ni-P catalyst deposited on the copper surface becomes progressively denser and smoother, thereby enhancing the corrosion resistance of the coating. However, beyond 5 V, the ‘coating’ begins to develop cracks, leading to a decline in corrosion resistance [37,38]. The maximum impedance value is observed at 5 V.

Figure 6c–f depict similar impedance characteristics. It can be observed that variations in deposition time and temperature result in peaks in the data. The analysis process is similar to that used for the effects of electrodeposition voltage; to avoid repetition, it will not be elaborated here. Under different deposition times and temperatures, the maximum impedances were obtained at 2 min and 30 °C, respectively. The equivalent electrical circuit (EEC) shown in Figure 6f (bottom left) was designed as the best fit to the experimental results [39,40]. The component definitions in the EEC model are as follows: Rs represents the solution resistance; CPE_1_ is the constant phase element associated with the loose and porous outer oxide layer in the high-frequency region; R_f_ is the resistance in parallel with CPE_1_; CPE_2_ is the constant phase element associated with the dense inner oxide layer; and R_ct_ represents the charge transfer resistance in the low-frequency region. The experimental data are represented by colored circles, and the fitting results are shown by solid lines. The parameter values of the experimental impedance spectra for Ni-P catalysts in hydrogen fuel cells, obtained from the EEC model, are listed in Table 2. From the data in Table 2, it can be seen that the charge transfer resistance R_ct_ reached its maximum value under the conditions of 5 V, 3 min, and 30 °C, respectively.

**Figure 7:**
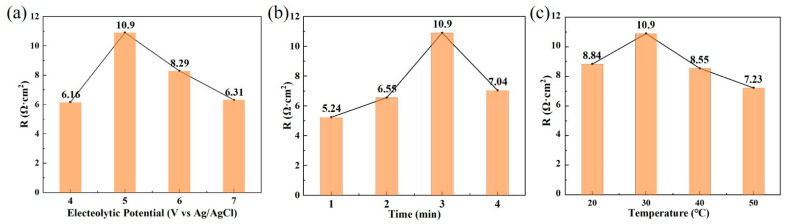
Bar chart of R_ct_ values of Ni-P/Cu catalysts under different preparation conditions: (**a**) voltage, (**b**) time, (**c**) temperature.

To more clearly illustrate the corrosion behavior of Ni-P catalysts under different deposition conditions, the charge transfer resistance (R_ct_), which represents the kinetic characteristics of the corrosion reaction, is plotted in Figure 7. It is evident that R_ct_ initially increases rapidly with increasing deposition voltage, then begins to decrease when the voltage exceeds 5 V. This trend indicates that when the Ni-P catalyst covers the copper surface, the initial corrosion rate decreases, as the “coating” provides good coverage for the substrate. However, as the deposition voltage further increases, R_ct_ decreases significantly, implying an increased corrosion rate due to the formation of numerous cracks on the catalyst surface. Figure 7b,c present similar results, showing that the maximum R_ct_ is obtained at 3 min and 30 °C, respectively. As described above, the optimal electrodeposition process parameters are 5 V, 3 min, and 30 °C.

**Figure 8:**
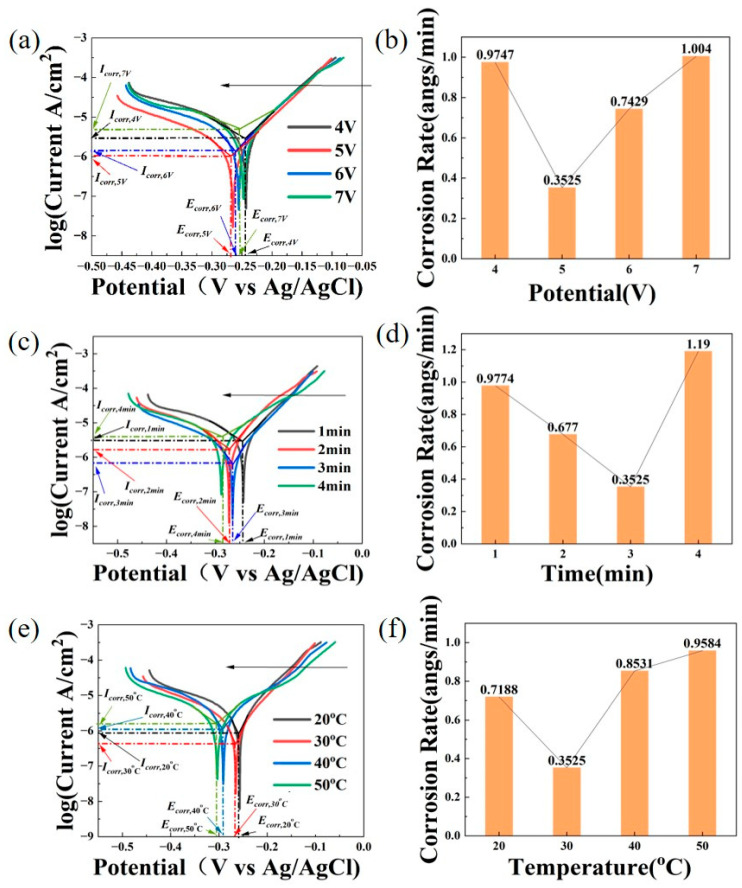
Polarization curves and corrosion rate diagrams of Ni-P/Cu catalysts under different preparation conditions. The black arrow indicates the scanning sequence. The dashed line indicates the corrosion current and corrosion potential, derived from the light-colored Tafel extrapolation curve, (**a**,**b**) Voltage, when electroplating temperature and time are 30 °C and 3 min; (**c**,**d**) time, when electroplating temperature and voltage are 30 °C and 5 V; (**e**,**f**) temperature, when electroplating voltage and time are 5 V and 3 min.

To further investigate the corrosion behavior of Ni-P catalysts in sodium borohydride fuel cells, Figure 8 presents the potentiodynamic polarization curve test results under varying deposition voltages, times, and temperatures. It is evident that the Ni-P catalyst did not exhibit a typical active-passive transition in the sodium borohydride-seawater mixed solution but primarily displayed active behavior, indicating continuous anodic active dissolution on the catalyst surface above the zero-current voltage [41,42]. As expected, with prolonged service time, the activity of the Ni-P catalyst gradually decreases, significantly shortening its service life. Furthermore, as the deposition voltage increases, the cathodic branch of Ni-P catalyst oxidation initially shifts in the negative direction and then in the positive direction, indicating that the corrosion dissolution current of the catalyst first decreases significantly and then sharply increases, reaching a minimum at 5 V. As shown in Figure 8a, the corrosion voltage (E_corr_) rapidly decreases with increasing deposition voltage before subsequently increasing. Specifically, at a deposition voltage of 4 V, E_corr_ is approximately 0.242 V, whereas at 5 V, it rapidly rises to 0.264 V. This behavior is primarily due to the catalyst layer becoming denser and smoother as the deposition voltage increases, resulting in a significant negative shift in the corrosion voltage of the electrochemical system. This finding is consistent with the OCP measurement results (Figure 5), confirming the accuracy of the polarization curve test. The E_corr_ and corrosion current density (I_corr_) were obtained from the polarization curves by Tafel extrapolation, as shown in Figure 8a. The corrosion rate can be calculated using the formula as:

CR = (M · I_corr_)/(nFS)

where CR is the corrosion rate (angs/min), and I_corr_ is the corrosion current density (µA·cm^−2^). M, n, F, and S represent the molar mass (g·mol^−1^), the number of electrons transferred (mol), the Faraday constant (C·mol^−1^), and the working electrode area (m^2^), respectively [43].

Figure 8b shows the corrosion rates of Ni-P catalysts under different electrodeposition voltages. It can be seen that the corrosion rate first decreases and then increases sharply, reaching a minimum at 5 V. Similarly, Figure 8c,e present the potentiodynamic experimental results of Ni-P catalysts under varying electrodeposition times and temperatures. The analysis process is similar and will not be repeated here. The results further demonstrate that anodic active dissolution continuously occurs on the Ni-P catalyst surface above the zero-current voltage. The corresponding corrosion rates are shown in Figure 8d,f, respectively. It can be observed that the minimum corrosion rates were obtained at 3 min and 30 °C, respectively, which is opposite to the trend of R_ct_ (Figure 7), because the smaller the R_ct_, the greater the electrochemical reaction activity.

**Figure 9:**
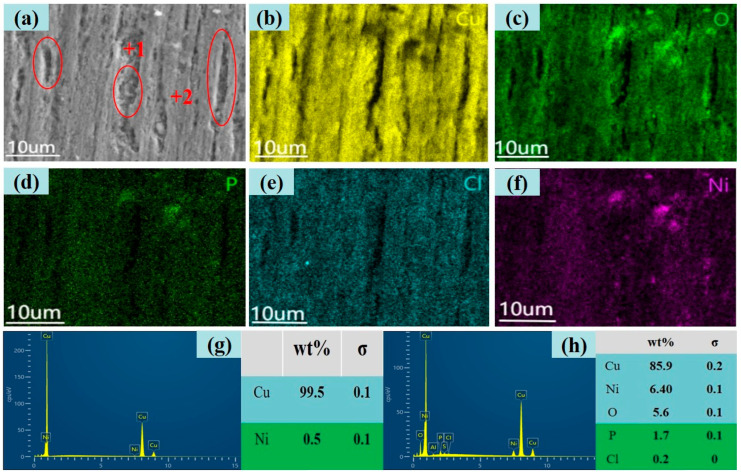
(**a**) SEM images of the catalyst after corrosion testing. The red circle indicates the selected region of the typical catalyst sample for comparative analysis. (**b**) EDS of Cu in the (+1) region, (**c**) EDS of O in the (+1) region, (**d**) EDS of P in the (+1) region, (**e**) EDS of Cl in the (+1) region, (**f**) EDS of Ni in the (+1) region, (**g**) EDS of the (+1) region, (**h**) EDS of the (+2) region.

After the potentiodynamic polarization experiments, the surface of the Ni-P/Cu catalyst was examined, as shown in Figure 9. It reveals that following corrosion, the catalyst on the copper surface had almost completely detached, and the copper substrate itself exhibited erosion characterized by striped grooves (Figure 9a). This indicates that corrosion adversely affects the catalyst’s service life, thereby impacting the operational efficiency of sodium borohydride fuel cells.

Two regions of the catalyst sample were selected for comparative analysis, namely the interior of the grooves and the copper surface. Pitting corrosion is evident on the catalyst surface in the (+1) region, with catalyst particles delaminated from the substrate in some areas. Figure 9g shows the elemental composition of the (+1) region, where the Cu content reaches 99.5% and the Ni content is only 0.5%, indicating severe corrosion in this area. The copper substrate has been exposed to the solution, and the catalyst layer has completely detached. The corresponding (+1) regions in Figure 9b–f also confirm this observation; the elemental distribution maps show that the (+1) region is almost entirely black, indicating that nearly no elements other than Ni and Cu remain. Examining the elemental content map of the (+2) region (Figure 9h), it is apparent that pitting corrosion in the (+2) region is confined to the catalyst layer. Catalyst elements Ni and P are still present in the (+2) region, along with O and Cl elements produced by corrosion. Furthermore, the elemental distribution in the (+2) region reveals that the corrosion depth did not penetrate deeply into the Cu plate but remained limited to the copper surface, with localized, non-uniform corrosion predominating.

A comparison between Figure 1 and Figure 9 reveals that as corrosion intensified, Ni and P gradually dissolved into the electrolyte, causing their concentrations to decrease rapidly. If corrosion continues with extended testing time, the catalyst will detach, significantly reducing both the catalyst’s service life and the internal operational efficiency of the hydrogen fuel cell.

**Figure 10:**
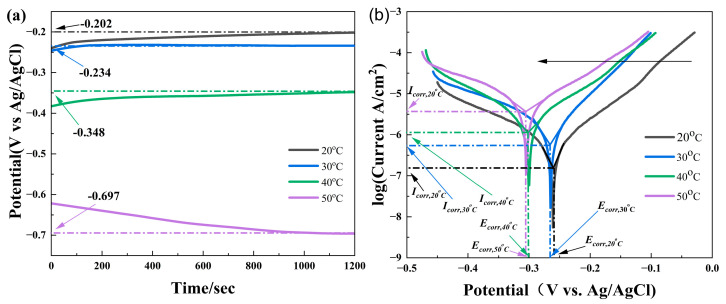
The OCP (**a**) and polarization curves (**b**) of the Ni-P/Cu catalyst under different solution temperatures of seawater. The coating preparation conditions were 5 V, 3 min, and 30 °C.

Based on the research above, we established the optimal catalyst preparation conditions as a deposition voltage of 5 V, a deposition time of 3 min, and a deposition temperature of 30 °C. Since the operating environment of fuel cells is influenced by temperature and chloride ion concentration, corresponding electrochemical corrosion studies were conducted. As shown in Figure 10, the OCP diagram of Ni-P/Cu catalysts at different seawater temperatures indicates that the OCP gradually increased over time before stabilizing. This increasing trend suggests that redox reactions occurred on the coating surface, gradually establishing a stable potential state. The initial open circuit potentials varied significantly at different temperatures; from a thermodynamic perspective, higher temperatures result in more negative open circuit potentials and greater susceptibility to corrosion. Figure 10 also presents the polarization curves of Ni-P/Cu catalysts at different temperatures. The corrosion current I_corr_ obtained through Tafel fitting reflects the corrosion rate of the material, with larger values indicating faster corrosion. At 50 °C, the potential was the most negative and I_corr_ was the highest, indicating the greatest susceptibility to corrosion.

**Figure 11:**
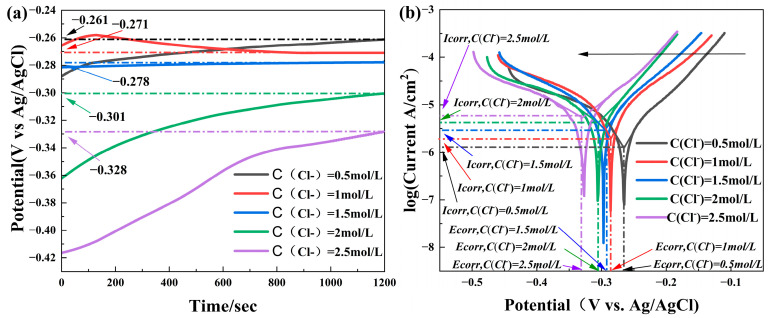
The OCP (**a**) and polarization curves (**b**) of the Ni-P/Cu catalyst under different chloride ion concentrations in simulated seawater. The coating preparation conditions were 5 V, 3 min, and 30 °C.

By varying the conditions of the simulated seawater solution, the OCP profiles of Ni-P/Cu catalysts at different chloride ion concentrations were obtained, as shown in Figure 11a. It is evident from the figure that the increasing trend in OCP reflects redox reactions occurring on the coating surface, eventually reaching a stable state. The initial open circuit potentials varied significantly under corrosion environments with different chloride ion concentrations. From a thermodynamic perspective, higher chloride ion concentrations result in more negative open circuit potentials and increased susceptibility to corrosion. The polarization curves of Ni-P/Cu catalysts at various chloride ion concentrations are presented in Figure 11b. Considering both E_corr_ and I_corr_ comprehensively, higher chloride ion concentrations lead to more negative E_corr_ and larger I_corr_. At a chloride ion concentration of 2.5 mol/L, the potential was the most negative and I_corr_ was the highest, indicating the greatest susceptibility to corrosion.

**Figure 12:**
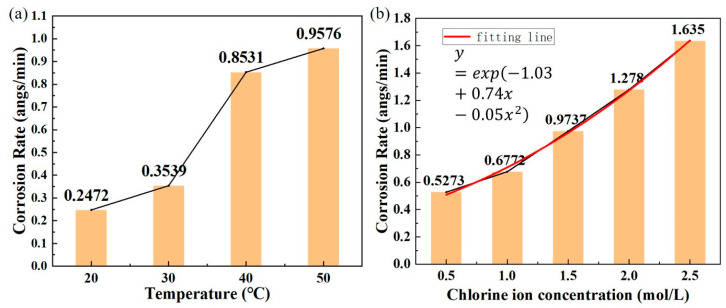
Corrosion rate curves of Ni-P/Cu catalysts under different corrosion conditions: (**a**) temperature, (**b**) chloride ion concentration.

Based on the OCP profiles comparison in Figure 10a and Figure 11a, the OCP curves corresponding to chloride ion concentrations exhibit greater fluctuations and lower values. This indicates that the presence of chloride ions increases the electrochemical activity of the catalyst and enhances the corrosion tendency of the catalyst layer. From the images in Figure 10b and Figure 11b, along with the corrosion rate formula, the corrosion rates of the catalysts at different temperatures and chloride ion concentrations were determined and plotted, as shown in Figure 12. It can be observed that chloride ions have a strong promoting effect on the corrosion rate of the catalyst.

**Figure 13:**
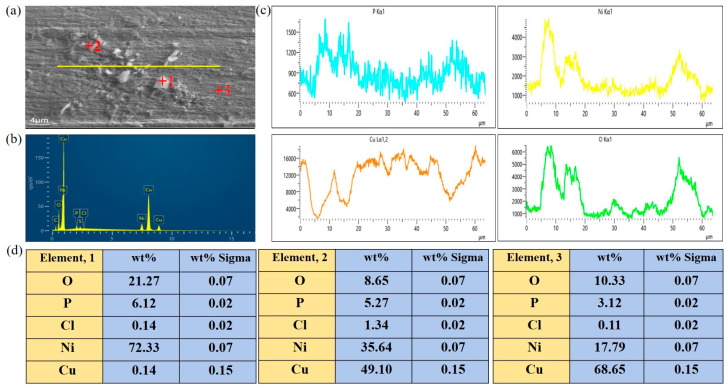
(**a**) SEM images obtained after corrosion testing of the catalyst in a solution with a CL ion concentration of 2.5 mol/L; (**b**) analysis of elements and valence states in catalysts; (**c**) fluctuations in elemental content as scanned along the yellow line in (**a**); (**d**) proportion of element quality in regions (+1), (+2), (+3).

Further investigation into the corrosion mechanism of chloride ions on the catalyst is conducted. Figure 13 illustrates the morphology and elemental composition of the catalyst after corrosion under a chloride ion concentration of 2.5 mol/L. Figure 13b shows the elements present in the catalyst and their possible oxidation states. By scanning the elements from left to right along the yellow line in Figure 10a and combining this with Figure 13d, it is observed that in the (+1) region, Ni and P account for 72.33 wt% and 6.12 wt%, respectively, while copper constitutes only 0.14 wt%, indicating that the catalyst coating remains well-preserved. In contrast, in the (+2) and (+3) regions—particularly the more severely affected (+3) region—the Ni and P contents drop significantly to 17.79 wt% and 3.12 wt%, respectively, whereas copper increases to 68.65 wt%, suggesting severe catalyst detachment and exposure of the substrate. These findings demonstrate that chloride ion corrosion in seawater affects the catalyst through pitting corrosion, exposing the substrate and thereby reducing the catalyst’s efficiency and service life.

**Figure 14:**
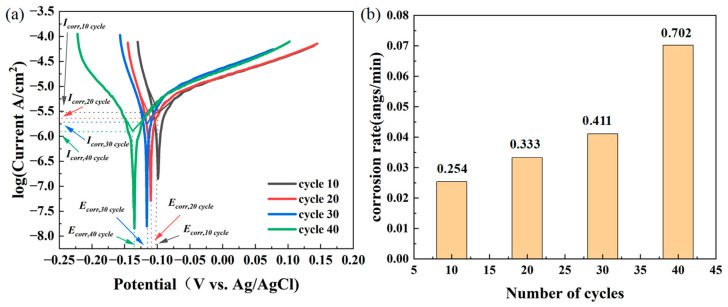
Study on long-term stability of Ni-P/Cu catalyst prepared under conditions of 5 V, 30 °C and 3 min: (a) different cycles of polarization curve tests, (b) variations in corrosion rate.

Figure 14 displays the polarization curves of the Ni-P catalyst after various cycles; the catalyst was prepared under conditions of 5 V, 30 °C and 3 min. It is seen that the anodic branches shift to the positive direction with increasing cycles (Figure 14a), implying decreasing corrosion resistance after recycling. The corrosion current density (I_corr_) was obtained by Tafel extrapolation to calculate the corrosion rate, as shown in Figure 14b. We can see that as the number of cycles increased from 10 to 40, the corrosion rate increased from 0.254 angs/min to 0.702 angs/min. This result further indicates that severe corrosion of the catalyst occurred in the NaBH_4_ seawater fuel cell. In summary, there is a trade-off between catalyst activity and corrosion resistance; that is, the better the catalytic performance, the faster the corrosion and the shorter the lifespan. Therefore, catalyst selection needs to balance both activity and corrosion resistance.

**Figure 15:**
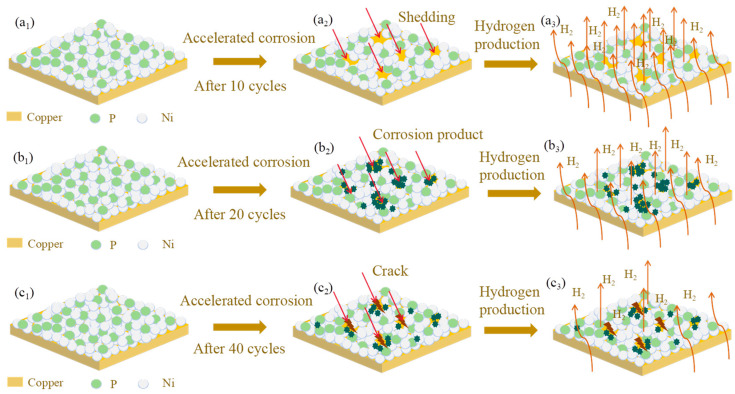
A schematic of the corrosion mechanism and hydrogen evolution process of the Ni-P/Cu catalyst in NaBH_4_ seawater solution after different cycles, (**a1**–**a3**) Schematic after 10 cycles, (**b1**–**b3**) Schematic after 20 cycles, (**c1**–**c3**) Schematic after 40 cycles.

Figure 15 shows a schematic of the corrosion mechanism and hydrogen evolution process of the Ni-P/Cu catalyst in NaBH_4_ seawater solution after different cycles. As clearly shown in the figure, with the number of cycles increasing from 10 to 40, the Ni-P catalyst begins to corrode and detach after prolonged immersion in NaBH_4_ seawater (c_1_, c_2_, c_3_) [44]. Simultaneously, the presence of chloride ions in the seawater causes electrochemical corrosion and dissolution of the Ni-P catalyst. These two factors lead to the gradual deactivation of the catalyst and cracking of the coating surface, resulting in decreased catalytic performance and a reduced hydrogen evolution rate (a_3_, b_3_, c_3_). With prolonged use, the sodium borohydride fuel cell will eventually fail due to corrosion, cracking, and detachment. Therefore, the catalyst selection must meet certain corrosion resistance requirements [45].

## 4. Summary and Conclusions

In this study, Ni-P catalysts were electrodeposited onto copper sheet surfaces, and the effects of deposition voltage, temperature, and time on the corrosion resistance of Ni-P/Cu catalysts in NaBH_4_ fuel cells were investigated. We found that as the deposition voltage increased, the coverage of the Ni-P catalyst on the copper surface gradually improved; however, excessive voltage caused cracking of the catalyst layer and detachment of catalyst particles. Electrochemical corrosion tests demonstrated that the corrosion resistance and catalytic efficiency peaked at a deposition voltage of 5 V. The structure of the catalyst has a significant effect on its performance. After determining the optimal deposition voltage, similar methods were used to study the effects of deposition time and temperature. The results indicated that the optimum preparation conditions of the catalyst were a deposition voltage of 5 V, a time of 3 min and a temperature of 30 °C. Seawater temperature and chloride ion concentration significantly affected corrosion rates. When the temperature increased from 20 °C to 50 °C, the corrosion rate of the Ni-P catalyst increased gradually with the increasing temperature. When the chloride ion concentration increased from 0.5 mol/L to 2.5 mol/L, the corrosion rate of the Ni-P catalyst increased exponentially. Therefore, the chloride ion concentration has a more significant effect on the corrosion resistance of the Ni-P catalyst than the temperature. Furthermore, the study found that chloride ion corrosion primarily affects the catalyst through pitting corrosion. By optimizing these process parameters, we can better predict the service life of Ni-P/Cu catalysts.

## Figures and Tables

**Table 1:** Detailed plating parameters of reagents and conditions for preparing the Ni-P catalyst supported on nickel foam.

Reagents and Conditions	Plating Parameters
NaH_2_PO_2_·H_2_O	0.075 mol/L
C_6_H_5_Na_3_O_7_·2H_2_O	0.035 mol/L
H_3_BO_3_	0.4 mol/L
NiSO_4_·2H_2_O	0.05 mol/L
pH	9.0 ± 0.2
Electroless plating temperature	20 °C, 30 °C, 40 °C, 50 °C
Corrosion test temperature	20 °C, 30 °C, 40 °C, 50 °C
Deposition time	1 min, 2 min, 3 min, 4 min
Deposition voltage	4 V, 5 V, 6 V, 7 V

**Table 2:** Parameter values obtained from EEC models on the experimental impedance spectra.

Factor	Factor	R_s_ (Ω·cm^2^)	Q_r_ (F·cm^−2^)	n_r_	R_r_ (Ω·cm^2^)	Q_dl_ (F·cm^−2^)	n_dl_	R_ct_ (Ω·cm^2^)
Voltage (V)	4	6.82	2.30 × 10^−4^	0.33	1410	1.25 × 10^−4^	0.89	6.16 × 10^3^
	5	6.67	6.88 × 10^−5^	0.40	3955	6.15 × 10^−5^	0.92	1.09 × 10^4^
	6	6.37	1.72 × 10^−4^	0.87	781	3.45 × 10^−4^	0.36	6.31 × 10^3^
	7	5.72	1.65 × 10^−4^	0.88	568	2.63 × 10^−4^	0.33	8.29 × 10^3^
Time (min)	1	5.80	2.70 × 10^−4^	0.83	2823	6.41 × 10^−4^	0.58	5.24 × 10^3^
	2	6.20	1.21 × 10^−4^	0.88	671	1.28 × 10^−4^	0.47	6.55 × 10^3^
	3	6.67	6.88 × 10^−5^	0.40	395	6.15 × 10^−5^	0.92	1.09 × 10^4^
	4	6.33	1.02 × 10^−4^	0.32	785	1.27 × 10^−4^	0.96	7.04 × 10^3^
Temp (°C)	20	6.06	1.56 × 10^−4^	0.85	253	1.26 × 10^−4^	0.36	8.84 × 10^3^
	30	6.67	6.88 × 10^−5^	0.40	395	6.15 × 10^−5^	0.42	1.09 × 10^4^
	40	6.26	6.98 × 10^−5^	0.39	407	1.07 × 10^−4^	0.92	8.55 × 10^3^
	50	6.27	9.68 × 10^−5^	0.89	1837	9.68 × 10^−4^	0.36	7.23 × 10^3^

## Data Availability

The original contributions presented in this study are included in the article. Further inquiries can be directed to the corresponding author.
